# Your pain is not mine: A critique of clinical empathy

**DOI:** 10.1111/bioe.12980

**Published:** 2021-12-12

**Authors:** Eugenia Stefanello

**Affiliations:** ^1^ Department of Philosophy, Sociology, Education and Applied Psychology (FISPPA) University of Padova Padova Italy

**Keywords:** clinical empathy, doctor–patient relationship, emotional resonance, epistemic injustice, paternalism, perspective‐taking

## Abstract

Both in mainstream culture and in bioethical literature, there is a general agreement on the absolute positive value assigned to empathy in healthcare settings. Thanks to its two components—affective and cognitive—clinical empathy should allow physicians to be emotionally affected by the experiences of their patients, and at the same time, to imagine their situations in order to gain a deeper understanding and implement a ‘tailored’ approach to care. So, it seems that good physician has become synonymous with empathetic physician. However, while acknowledging its numerous benefits, I will argue that clinical empathy seems to harbour some dark sides. First, the affective component of clinical empathy (i.e. emotional resonance) is responsible for its partial nature and can lead to cognitive and moral distortions. Moreover, it can lead healthcare providers to negative psychological states, such as burnout and personal distress. Second, the cognitive component of empathy can be problematic as well: perspective‐taking is a far more difficult task than it is ordinarily thought to be. I will also try to demonstrate that accessing the inner world of others is neither possible nor desirable since this operation can result in undermining the patient's agency. Third, clinical empathy can become a tool that disguises the power imbalance between patients and doctors, and this can reinforce an elitist and paternalistic conception of the clinical encounter. Furthermore, the disregard for the influence that the sociocultural context has on the clinical relationship can amplify and promote instances of epistemic injustice perpetuating discriminatory and unfair dynamics.

## 1 INTRODUCTION

While empathy has mostly been considered indispensable to a satisfactory doctor–patient relationship,^1^ some critical voices have recently emerged both regarding its role in the clinical encounter and in morality in general.^2^ Clinical empathy sceptics doubt that an empathic understanding of patients is possible without either the risk of imposing the physician's interpretative categories upon others' experience^3^ or completely disregarding the sociocultural situatedness of the subjects involved in the empathic relationship.^4^ Yet, despite these concerns, there seems to persist a largely shared belief among both professionals and the general population that for healthcare professionals to be considered good, they need to be empathetic. Accordingly, it is difficult to find a medical school programme that does not include empathy as one of its educational goals.^5^ Empathy also appears to be the ground on which the relationship between healthcare authorities and the public must rest, especially during times of crisis. For instance, the Centers for Disease Control and Prevention (CDC) places ‘empathy and caring’^6^ at the top of the list of factors that make communication credible and trustworthy in the eyes of journalists and communities during a health emergency. An example of this is the fact that being ‘respectful, polite and empathetic’^7^ is the first piece of advice that the World Health Organization (WHO) gives to healthcare professionals in order for them to communicate effectively with patients who have contracted COVID‐19.

However, while acknowledging the numerous benefits of clinical empathy, I will argue that the empathic phenomenon can have some dark sides that are worth discussing. First, I will show, from a neuroethical standpoint, that the affective component of clinical empathy (i.e. emotional resonance) is responsible for its partial nature and can lead to cognitive and moral distortions. Moreover, it can lead healthcare providers to negative psychological states, such as burnout and personal distress. Second, I will argue, based on an epistemological standpoint, that the cognitive component of empathy can be problematic as well: perspective‐taking is a far more difficult task than it is ordinarily thought to be. I will also try to demonstrate that accessing the inner world of others is neither possible nor desirable since this operation can result in undermining the patient's agency. Third, I will discuss some moral issues that clinical empathy poses to the doctor–patient relationship. In particular, when the patient's experience is seen as a sort of ‘territory to be conquered’ by the doctor, clinical empathy can become a tool that disguises the power imbalance between patients and doctors, and this can reinforce an elitist and paternalistic conception of the clinical encounter. Furthermore, the disregard for the influence that the sociocultural context has on the clinical relationship can amplify and promote instances of epistemic injustice perpetuating discriminatory and unfair dynamics.

## 2 TWO SIDES OF THE SAME COIN: AFFECTIVE AND COGNITIVE EMPATHY

In the last 20 years, clinical empathy has slowly but steadily replaced detached concern as the core value in medical care: ‘physicians must connect with rather than detach from their patients, especially their emotional states in order to provide genuine healing’.^8^ This paradigm shift has forced healthcare professionals and bioethicists to reflect on the nature of empathy.

The definition of empathy in general and clinical empathy in particular is a thorny issue. Nevertheless, there is a general consensus on the existence of two components of empathy that are not only conceptually and phenomenally distinct but also involve different neural networks:^9^ affective empathy and cognitive empathy. Notably, even though it is possible to identify these two components, it does not mean that they do not interrelate or cannot operate simultaneously.

Affective empathy encompasses different phenomena with various degrees of complexity, such as affective empathy proper, sympathy, emotional contagion or personal distress, and generally consists of ‘a range of emotional responses we can have to what others feel or the situation they are in’.^10^ By contrast, cognitive empathy is usually regarded as ‘the capacity to understand another person's state of mind from her perspective’.^11^ Here, the empathizer consciously shifts her perspective to ascribe mental states or emotional experiences to the other person.

The bioethical reflection on clinical empathy has embraced this taxonomy and consequently developed a variety of accounts considering both components of empathy. In particular, Jodi Halpern has provided a nuanced and rich definition of clinical empathy in which its hybrid nature is perfectly highlighted: it is ‘the ability to resonate [that] allows the curious physician to use her imagination. The imaginative use of the physician's affects provides a framework, an organizing context for understanding the patient's particular experience’.^12^ So, healthcare professionals should always remain emotionally available to be affected by and open to the particular existential experience of their patients, and at the same time, they should imagine how their patients feel about their conditions of illness, and this will allow them to understand the specific situation of each individual patient. Emotional openness and imaginative curiosity should improve the overall quality of the interaction of care. In this sense, empathy strengthens the doctor's communication skills and therefore enhances—and does not compromise—the reliability of the diagnostic process. In this respect, it proves to be an extremely useful heuristic tool since it stimulates patients to provide crucial information that would otherwise have been unavailable to the doctor.^13^ A deeper understanding of the condition of patients should enable healthcare professionals to create a stronger and more trustworthy clinical relationship. This, in turn, should lead to an increase in patient satisfaction and consequently, to a higher adherence rate to the doctor's therapeutic guidelines.^14^

So, clinical empathy appears to be particularly appealing as it helps clinicians implement a ‘tailored’ approach to care. By recognizing and valuing the uniqueness of each patient's existential perspective, the empathic doctor should be able to develop a highly specific therapeutic plan. Yet, in order for doctors to properly imagine how their patients feel and therefore have a better understanding of their patients' conditions, they must experience, to some extent at least, the same emotional states. A certain level of emotional isomorphism between doctors and patients is required, and it can be achieved through emotional resonance, which is defined as the ‘spontaneous affect that is similar to another's affect, such as feeling anxious around an anxious person’.^15^ Although Halpern assigns a preliminary role to emotional resonance, I believe that its involvement in clinical empathy raises some issues: as I will try to show below, the fact that physicians are required to share the same emotional states as their patients, even if just at the beginning of the process, can be harmful both to the patient and the healthcare professionals. At the same time, I think that advocating for a purely cognitive form of clinical empathy is not an effective solution either; on the contrary, it can be as problematic as affective resonance‐based empathy. Finally, even without these concerns, clinical empathy can still have troubling consequences on the already asymmetrical distribution of power and the unequal level of epistemic credibility in the doctor–patient relationship. In the next sections, I will try to articulate and discuss these criticisms.

## 3 THE DARK SIDE OF EMPATHY

Far from being the *panacea* for all problems, clinical empathy seems to harbour some dark sides. In order to demonstrate this, I will use a threefold argument: the first argument is of a neuroethical nature and addresses the problems of affective empathy, the second discusses epistemological questions related to cognitive empathy and the third investigates moral issues arising from clinical empathy in general.

### 3.1 The neuroethical argument

From a neuroethical perspective, there are two main problems with the affective component of clinical empathy: first, it can lead to cognitive and moral distortions; second, it can lead healthcare providers to negative psychological states, such as burnout and personal distress.

With regard to the first aspect, the fact that clinical empathy is based on emotional resonance is extremely problematic because the empathetic subject can be affected by the so‐called ‘similarity bias’,^16^ which is the tendency for the empathizer to empathize better and deeper with those who resemble and are near and dear to her. The results of several neuroethical studies confirm the existence of this prejudice. For example, Xu et al. report, ‘whereas painful stimulations applied to racial in‐group faces induced increased activations in the ACC and inferior frontal/insula cortex in both Caucasians and Chinese, the empathic neural response in the ACC decreased significantly when participants viewed faces of other races’.^17^

It seems that the degree of empathy experienced by the tested subject is directly proportional to the degree of emotional proximity, spatial proximity and temporal proximity. These findings reflect and explain some aspects of our behaviour that are rather common, if not universally shared. It is because of this emotional proximity that we empathize more with our friends and family, people that are usually near and dear to us. The element of spatial proximity is also very important and may explain why we usually care significantly more about the fate of people who are from our neighbourhoods, our cities and our countries, despite the fact that we do not know them personally. Lastly, temporal proximity is a key feature in the ability to empathize with others. Consider, for example, how difficult it is to imagine and care about future generations—human beings that are not even born yet—in regard to the catastrophic effects of the climate emergency.

Therefore, ‘empathy is neither universal nor an automatic response’.^18^ On the contrary, the selective nature of empathy stems from the fact that our empathic responses are strongly modulated by group membership, and the parameters that draw the boundaries of the group can vary considerably ranging from race, gender and political affiliation to football team preference.^19^ In this respect, one could object that we have developed strategies to mitigate these biased mechanisms. The problem is that these strategies seem unable to overcome the fact that ‘we can empathize with members of the out‐group but only by making their similarities salient’.^20^ The ‘local’ character of empathy seems inescapable, and the only way to reduce its negative effects would be to minimize—if not eliminate—the peculiarities of the other that must become, at any cost, similar to us. So, the only way by which empathy could work would be via the reduction of the differences between the empathizer and the subject of empathy.

However, this seems especially problematic in clinical practice where doctors and patients usually have very dissimilar perspectives, values, life experiences, social and ethnic backgrounds, and so on. It is precisely because of this similarity bias that doctors who use clinical empathy can make cognitive errors and risk making morally wrong choices. Empathy becomes particularly tricky when the moral situation requires the use of justice and equality criteria because ‘empathic reactions are inherently linked to partiality. This partiality necessitates a framework of justice principles to counter empathy's biasing effects and keep social allocation behaviours in check’.^21^ This correlation between injustice and empathy is corroborated by Decety and Yoder: ‘interestingly, and counterintuitively, the emotional facet of empathy (…) was not significantly correlated with justice either for oneself or for the other. Personal distress also did not improve the model for other‐oriented justice sensitivity’.^22^

These findings are strongly in contrast with the assumption that morality requires empathy, which is a position largely endorsed by both the mainstream culture and within the academic debate.^23^ The premise for this argument is that human beings are intrinsically selfish. The only way through which these selfish tendencies can be overcome is by making the condition of others relevant to one's individual situation. Only empathy, since it is able to connect with the emotional experiences of others, can ‘guide us to treat others as we treat ourselves and hence expands our selfish concerns to encompass other people’.^24^ The problem with this perspective is that it is absolutely necessary to feel the emotional states of others in order to make their situations relevant to us. However, feeling what others are feeling is extremely taxing and demanding for any empathizer, especially for healthcare providers who are exposed to incredible amounts of suffering, pain and death on a daily basis.

This leads us to the second issue: the emotional resonance element in clinical empathy can cause states of psychological distress in doctors, among which the most serious is burnout. Its consequences can be very dangerous: ‘deterioration in patient care, medical errors, substance abuse, interpersonal difficulties, depression and suicide’.^25^ In this respect, Lamothe et al. have found that ‘affective sharing without emotion regulation skills may be associated with personal distress, compassion fatigue and burnout, which would turn into decreasing empathic concern and pro‐social helping behaviour’.^26^ Gleichgerrcht and Decety have also found similar results: ‘it is possible that physicians who are most vulnerable to emotional distress and compassion fatigue, which may lead to emotional exhaustion, and a low sense of accomplishment, are those who have difficulties regulating their negative arousal’.^27^ Therefore, clinical empathy has to be regulated through a cognitive intervention. However, even with this cognitive regulation, ‘it is important to note that a modicum of negative arousal is necessary to help physicians attune to and empathically understand patients’ emotions'.^28^ So, it seems that some level of emotional fatigue is inevitable for the doctor who wants to be empathic.

### 3.2 The epistemological argument

One might say that since emotional resonance causes all of these problems, perhaps a purely cognitive concept of clinical empathy might be the answer we are looking for. Note that this position implicitly assumes that it is possible for healthcare professionals to switch one of the two components of clinical empathy on and off as if they were completely independent of one another, which in itself, is a controversial thesis to argue. Despite this, supporters of cognitive empathy claim that the desired benefits of clinical empathy, such as engaged communication and personalized care, can be achieved solely with perspective‐taking without having to deal with the downsides of emotional resonance.^29^ However, I am not convinced that perspective‐taking poses fewer challenges than emotional resonance, and I think that the attempt to understand and have access to the inner world of others is an extremely complicated process. When we discuss understanding others, we often seem to forget how baffling the complexity of our minds is: ‘another person's mind is one of the most complicated systems that any person will ever think about’.^30^ In fact, ‘neuroscientists calculate that a human brain could be in more possible brain states than there are elementary particles in the universe’.^31^

Clearly, the complexity itself does not impede the feasibility of this operation. Yet, when we engage in perspective‐taking, we are satisfied only if the understanding we have acquired is accurate. Most importantly, when others are trying to grasp our perspective, we want them to understand us as accurately as possible. This desire is even stronger if we are suffering physically, emotionally and mentally as occurs during an illness. In this regard, given how inherently complex, time‐consuming and relevant the understanding of the patient's perspective is, physicians might be tempted to use a sort of ‘fake empathy’,^32^ which, in the clinical context, can consist of a standardized set of expressions and procedures that tries to simulate, almost mechanically, genuine and accurate empathic understanding.^33^ However, I believe that it is not a valid option for intersubjective interactions and is especially counterproductive in the doctor–patient relationship for at least two reasons: first, it seems to be ineffective since it deliberately does not aim—and therefore it is not able—to obtain any information about the patient but merely mimics the process of understanding. Second, ‘fake empathy’ can compromise the clinical relationship because, if patients recognize it, it could cause an erosion of trust between them and the physician. Interestingly, the reaction triggered by the recognition of this purely performative empathy can be seen as similar to the uncanny valley effect, which consists of an immediate disgust and aversion toward those androids that too closely resemble human beings.^34^

Aside from the case of ‘fake empathy’, it is also possible that the empathic doctor simply may not be able to gain an accurate understanding of the patient's perspective, even though she is sincerely trying to empathize with her. While this inability may stem from the opacity of the other's perspective or an honest mistake of the empathizer^35^—which makes it different from ‘fake empathy’—I would argue that it can still be challenging because, if not addressed and corrected, it can have negative consequences for the clinical encounter. In fact, on the one hand, if diagnostic and therapeutic decisions are made on the basis of information derived from perspective‐taking, it should be accurate, on the other, the inaccuracy could cause misunderstandings as well as conflicts between doctors and patients.^36^

So, given its importance, is perspective‐taking the right strategy to attain accuracy? According to empirical evidence, not really. The widespread idea behind the benefits of perspective‐taking is fairly straightforward: putting oneself in the other's shoes seems to be an effective way to contrast our egotistical inclinations and at the same time, our tendencies to stereotypically categorize others' experiences. However, little attention has been directed at verifying that accuracy is *truly* attained through perspective‐taking. In this respect, Eyal et al. have found surprising results: ‘across nine experiments consisting of naturalistic tests of interpersonal accuracy—predicting a partner's preferences and opinions—we found that an explicit instruction to engage in perspective‐taking did not increase accuracy. If anything, it decreased accuracy’.^37^

However, I want to take the argument further: I will argue, in line with Peter Goldie's work, that perspective‐taking is not only the wrong tool for achieving accuracy when it comes to interpersonal understanding, but also that having full access to the inner world of others is neither possible nor desirable, particularly in the clinical encounter.

In this regard, Goldie argues that there are two types of empathy:

very roughly speaking, what I am against is what I call *empathetic perspective‐shifting*: consciously and intentionally shifting your perspective in order to imagine *being* the other person, and thereby sharing in *his or her* thoughts, feelings, decisions, and other aspects of their psychology. I am not against what I will call *in‐his‐shoes perspective‐shifting*: consciously and intentionally shifting your perspective in order to imagine what thoughts, feelings, decisions, and so on *you* would arrive at if you were in the other's circumstances.^38^

Goldie's empathetic perspective‐shifting essentially coincides with the imaginative component in Halpern's clinical empathy. In Goldie's opinion, the main problem is that it is impossible for an external subject to assume the same first‐person position taken by the agent himself: ‘only the agent himself can take his stance towards his own thoughts, decisions, and intentions’.^39^ It is possible to imagine being the other person only in what he calls ‘base cases’, in which a thin notion of agency is involved, that is, where the characteristics of the subject making a decision do not influence the decision itself. Therefore, in a base case, all the subjects who possess a *minimum* amount of rationality will formulate the same decision because the deliberation process is independent of the relevant aspects of the subject's character. Who you are does not matter; hence, the deliberative subjects are interchangeable.

On the contrary, in cases where a stronger notion of agency is involved, the attempt to take the first‐person perspective from another subject's point of view is bound to fail. In fact, in cases where a strong notion of agency is involved, the characteristics of the subject who decides, thinks and experiences emotions are crucial in the decision‐making process. I would argue that this aspect is particularly relevant here because the majority, if not all decisions that patients and doctors are called to make undoubtedly involve a strong sense of agency. How the subject sees and experiences the world, her values and priorities, her imaginative projects for the future, her past experiences and emotional history, are not only relevant but are also essential aspects in the deliberative process. There is an inherent specificity about clinical decision‐making that forces the existential condition of the subject to be taken into consideration as one of the most important aspects of the process.

Given the importance of a strong notion of agency in the clinical decision‐making process, Goldie's main thesis is even more relevant. He claims that when one tries to use the empathetic perspective‐shifting, there is a serious risk of usurping the agency of the subject with whom one empathizes.^40^ When empathizers try to assume the first‐person perspective, they will always risk abusing and mystifying the deliberative effort of the other. In this sense, the inner world of the other seems to be precluded. This does not mean that there is no space for communication and mutual understanding. The point is that we cannot—and must not—have the arrogance to place ourselves in the existential world of others from their point of view because not only can this be harmful to the other, but it is also epistemologically impossible. The exact reproduction of the inner world of the other person, which forms the unconscious background against which the deliberative process happens, is simply unattainable. It is too unique and too personal to be replicated by someone else. At best, we can try to imagine what *we* would have done or felt or thought in the same situation in which the other is.

### 3.3 The moral argument

Leaving aside the obstacles that we have encountered so far, I think that the majority of accounts of clinical empathy still fail to discuss how empathy can affect the power balance within the patient–doctor relationship. As Rebecca Garden says, even Halpern's comprehensive account tends to obscure the unequal distribution of power between the doctor and the patient: “where some might caution physicians against assuming that they can fully understand the experience of patients, Halpern argues that physicians should imagine ‘how it feels to have a certain illness, disability, or psychological injury’”.^41^ It seems that it is always possible for the doctor to penetrate and master the understanding of the existential world of the patient. While it is certainly true that sometimes this can be difficult and require more effort and time for the doctor, the difficulty of empathic labour does not imply its impossibility. Hence, for Halpern, the ability to empathically understand the patient simply becomes another skill that doctors are required to have. However, this way of defining empathy has an important consequence: it ‘situates the patient's experience squarely within the realm of the physicians' expertise’.^42^ So, the interpretative work of empathy seems to become an issue that is beyond the interest of the patient and becomes a concern exclusively for the doctor. Conversely, ‘in regards to the experience of pain and illness, the patient rather than the physician is the expert’.^43^ Recognizing that patients have a greater degree of knowledge and experience than their doctors about their own conditions, has the ability to return the power to the patients themselves. If doctors fail to understand that the inner world of the patient will always remain, in some respects, beyond their cognitive reach, we will face what can be called ‘the colonization problem’. When the patient's experience is seen as a sort of ‘territory to be conquered’, empathy becomes a tool that disguises the exercise of the doctor's power. Those who empathize have, quite literally, the experiences of others at their disposal, and they always have the power to modify and mystify them.

Furthermore, I believe that it is troubling to depict doctors as sort of omniscient beings. In this perspective, doctors can and should understand everything about their patients, from their physiological functions and malfunctions to the mental and emotional processes, even those that are more intimate and personal. Garden sees this danger too and warns us about the risk of empathy drifting toward elitism: physicians alone, in this elitist perspective, are able to understand and feel for others in pain because of their natural ability and their hard training. In this sense, the empathic encounter can always conceal the risk of an instrumental use of the suffering of others, where the ultimate goal becomes being satisfied with one's ability to understand the emotional experience of the other. Physicians should not use clinical empathy in order to ‘aestheticize illness’^44^ just because they have the power and the alleged skill to do so. Moreover, this elitist conception of clinical empathy reinforces a paternalistic behaviour toward patients: if physicians are morally and cognitively perfect beings, ‘patients should be awed, subdued and grateful for their treatment’.^45^ On the contrary, the doctor–patient relationship should be understood as horizontal and active, and the asymmetry between the two subjects—which will never be completely overcome—should be rebalanced as much as possible.

If it is true that patients are not ‘lands of conquest’ and that they will always remain in some aspects an unknowable otherness, it is equally important to point out that they are not atomized entities and that clinical encounters never take place in a vacuum. On the contrary, patients are always members of a community whose cultural and social background has a critical impact on their overall medical condition. For this precise reason, the empathetic physician needs not only to recognize the influence that the sociocultural context has on the doctor–patient relationship but also should develop ‘a context‐oriented action component [that] may speak more comprehensively to structural issues such as social determinants of health and power relations in the clinic’.^46^

The widespread absence of this context‐oriented attention in clinical empathy reflections can have worrisome consequences because it can amplify and promote instances of epistemic injustice. Epistemic injustice, defined by Miranda Fricker as ‘a wrong done to someone specifically in their capacity as a knower’,^47^ is ‘integrally related to social injustice’^48^ because it arises from biases, negative stereotypes and prejudices that can result in discriminatory behaviours. If we fail to acknowledge the ubiquity of negative stereotypes and prejudices, we might consequently fail to remove them as well as to grant the deserved epistemic credibility to other people's testimonies and experiences.

In this respect, it has been argued that epistemic injustice is particularly pervasive in healthcare settings for at least two reasons: on the one hand, patients ‘are often regarded as cognitively unreliable, emotionally compromised, or existentially unstable in ways that render their testimonies and interpretations suspect’.^49^ This suspicion can result in low or non‐existent epistemic credibility. In this sense, the lack of credibility depends on the ontological condition of patients themselves: the very fact of being ill inhibits their ability as knowers. On the other hand, when it comes to experiences of illness, both physical and mental, traditional ways of communicating are often unsuitable, and consequently, patients express themselves differently. This nonconformity with the propositional standard of communication can lead patients to suffer from a specific kind of epistemic injustice, namely hermeneutical injustice. Healthcare professionals may lack the appropriate interpretive resources to understand patients' experiences, and therefore, their narratives can be disqualified from an epistemic perspective. In this context, when patients belong to already socially marginalized groups or suffer from stigmatized pathologies, the prejudices in which epistemic injustice is rooted are going to be inevitably worsened. Socially marginalized groups can experience what Kidd and Carel call a ‘tracker prejudice, as the prejudices imposed by the negative stereotype track them through different domains of the social world’,^50^ including the clinical one. Similarly, the epistemic dignity of patients who suffer from stigmatized pathologies can be denied because of the prejudice that illness is ‘an expression of morality’,^51^ and therefore, these patients are not worthy of epistemic credibility and must be, in some sense, punished for their ‘moral failures’.

This vicious circle between social injustice, prejudices and epistemic injustice proves how critical it is for all clinical empathy accounts to recognize not only the unequal level of epistemic privilege but also the importance of situating the doctor–patient relationship within the sociocultural context of patients in order to recognize and potentially reduce the harm of negative prejudices that perpetuate the dynamics of epistemic injustice. At the same time, physicians must always bear in mind that the cultural and social differences between them and their patients are not simply some temporary obstacles that they are sooner or later going to overcome or some sort of empirical data that they can factor in during the decision‐making process. Instead, they need to find the right balance between the required understanding of the context and the awareness that this distance is never going to be completely bridged. Undoubtedly, achieving this balance is an onerous process whose difficulty directly depends on the ambiguous nature of empathy itself, which ‘is always perched precariously between gift and invasion’.^52^ However, it is important to clarify that the point of discussing the perils of epistemic injustice is to unearth and reflect on ‘certain policies, practices and cultural norms within modern healthcare practice [which] are liable to generate epistemic injustice’^53^ and not to imply that every healthcare professional will unavoidably perpetrate epistemically unjust behaviours.

In conclusion, the suffering of others, their inner worlds and their existential experiences are undoubtedly important to clinical practice and to the therapeutic process itself. Nevertheless, healthcare professionals must be aware of the ethical problems that clinical empathy poses especially regarding the concealment of the power imbalance and the influence of the sociocultural context.

## 4 CONCLUSION

Empathy is considered essential to clinical practice today more than ever. For this reason, healthcare professionals are always encouraged to develop and enhance their empathic skills. However, I have tried to demonstrate that this stance can be extremely problematic both from a theoretical and practical point of view. Clinical empathy is a multifaceted phenomenon with positive and negative sides, and ignoring the latter can be counterproductive both for healthcare professionals and patients.

By pointing out these difficulties, I do not aim to imply that empathy must be disregarded completely; on the contrary, I have tried to argue that, precisely because of its relevance, a critical and comprehensive approach is required. Clinical empathy deserves to be discussed in a way that takes into account its complexity, and this means that its shortcomings need to be addressed with the same accuracy as its successes.

I believe that the underlying problem of the debate about empathy is its alleged exclusivity when it comes to the clinical encounter: the idea that the interaction between doctors and patients can be subsumed in its entirety by the empathic relationship is not only wrong but can also inhibit further discussion. Clinical empathy is not a one‐fits‐all solution, and that is why other affective and cognitive tools should be thoroughly evaluated as possible alternatives or additions to improve the doctor–patient relationship. In doing so, perhaps we may find that empathy is not the answer to all our questions.

## CONFLICT OF INTEREST

The author declares no conflict of interest.

## Footnotes

1 Spiro, H. (2009). The practice of empathy. *Academic Medicine, 84*(9), 1177–1179; Gelhaus, P. (2012). The desired moral attitude of the physician: (I) empathy. *Medicine, Health Care and Philosophy, 15*, 103–113.

2 Prinz, J. (2011). Is empathy necessary for morality? In A. Coplan & P. Goldie (Eds.), *Empathy: Philosophical and psychological perspectives* (pp. 211–229). Oxford University Press; Bloom, P. (2017). Empathy and its discontents. *Trends in Cognitive Sciences, 21*(1), 24–31.

3 Slaby, J. (2014). Empathy's blind spot. *Medicine, Health Care and Philosophy, 17*, 249–258.

4 Pedersen, R. (2008). Empathy: A wolf in sheep's clothing? *Medicine, Health Care and Philosophy, 11*, 325–335.

5 Pedersen, R. (2010). Empathy development in medical education: A critical review. *Medical Teacher, 32*, 593–600.

6 Tumpey, A. J., Daigle, D., & Nowak, G. (2018). *Communicating during an outbreak or public health investigation*. Centers for Disease Control and Prevention. https://www.cdc.gov/eis/field-epi-manual/chapters/Communicating-Investigation.html#anchor_1543608947

7 World Health Organization. (2020). *The COVID‐19 risk communication package for healthcare facilities*. https://www.who.int/docs/default-source/coronaviruse/risk-communication-for-healthcare-facilities.pdf?sfvrsn=2a5b0e0b_2

8 Marcum, J. A. (2008). *Humanizing modern medicine. An introductory philosophy of medicine*. Springer, p. 266.

9 Hein, G., & Singer T. (2008). I feel how you feel but not always: The empathic brain and its modulation. *Current Opinion in Neurobiology, 18*, 153–158.

10 Maibom, H. L. (2017). Affective empathy. In H. L. Maibom (Ed.), *The Routledge handbook of philosophy of empathy* (pp. 22–32). Routledge, p. 22.

11 Spaulding, S. (2017). Cognitive empathy. In H. L. Maibom (Ed.), *The Routledge handbook of philosophy of empathy* (pp. 13–21). Routledge, p. 13.

12 Halpern, J. (2001). *From detached concern to empathy. Humanizing medical practice*. Oxford University Press, p. 85.

13 Coulehan, J. L., Platt, F. W., Egener, B., Frankel, R., Lin, C. T., Lown, B., & Salazar, W. H. (2001). “Let me see if I have this right…”: Words that help build empathy. *Annals of Internal Medicine, 135*(3), 221–227.

14 Halpern, J. (2014). From idealized clinical empathy to empathic communication in medical care. *Medicine, Health Care and Philosophy, 17*(2), 301–311.

15 Halpern, op. cit. note 12, p. 79.

16 Prinz, J. (2011). Against empathy. *The Southern Journal of Philosophy, 49*(1), 214–233, 227.

17 Xu, X., Zuo, X., Wang, X., & Han S. (2009). Do you feel my pain? Racial group membership modulates empathic neural responses. *The Journal of Neuroscience, 29*(26), 8525–8529, 8525; Gutsell, J. N., & Inzlicht, M. (2010). Empathy constrained: Prejudice predicts reduced mental simulation of actions during observation of outgroups. *Journal of Experimental Social Psychology, 46*(5), 841–845.

18 Zaki, J., & Cikara, M. (2015). Addressing empathic failures. *Current Directions in Psychological Science, 24*(6), 471–476, 471.

19 Hein, G., Silani, G., Preuschoff, K., Batson, C. D., & Singer, T. (2010). Neural responses to ingroup and outgroup members' suffering predict individual differences in costly helping. *Neuron, 68*(1), 149–160.

20 Prinz, op. cit. note 16, p. 228.

21 Decety, J., & Yoder, K. J. (2016). Empathy and motivation for justice: Cognitive empathy and concern, but not emotional empathy, predict sensitivity to injustice for others. *Society for Neuroscience, 11*(1), 1–14, 3.

22 Ibid: 10.

23 Simmons, A. (2014). In defense of the moral significance of empathy. *Ethical Theory and Moral Practice, 17*, 97–111.

24 Bloom, P. (2016). *Against empathy. The case for rational compassion*. Vintage, p. 21.

25 Lamothe, M., Boujut, E., Zenasni, F., & Sultan, S. (2014). To be or not to be empathic: The combined role of empathic concern and perspective taking in understanding burnout in general practice. *BMC Family Practice, 15*(15), 1–7, 1.

26 Ibid: 5.

27 Gleichgerrcht, E., & Decety, J. (2013). Empathy in clinical practice: How individual dispositions, gender, and experience moderate empathic concern, burnout, and emotional distress in physicians. *PLoS ONE, 8*(4), 1–12, 1.

28 Ibid: 11.

29 Schwan, D. (2018). Should physicians be empathetic? Rethinking clinical empathy. *Theoretical Medicine and Bioethics, 39*(5), 347–370.

30 Eyal, T., Epley, N., & Steffel, M. (2018). Perspective mistaking: Accurately understanding the mind of another requires getting perspective, not taking perspective. *Journal of Personality and Social Psychology, 114*(4), 547–571, 547.

31 Ibid: 547.

32 Morton, A. (2017). Empathy and imagination. In H. L. Maibom (Ed.), *The Routledge handbook of philosophy of empathy* (pp. 180–189). Routledge, p. 181.

33 Gardner, C. (2015). Medicine's uncanny valley: The problem of standardizing empathy. *Lancet, 386*(9998), 1032–1033, 1033.

34 Ibid: 1032.

35 Spaulding, S. (2016). Mind misreading. *Philosophical Issues, 26*(1), 422–440.

36 I would like to thank one of the anonymous reviewers for helping me clarify this point.

37 Eyal et al., op. cit. note 30, p. 561.

38 Goldie, P. (2011). Anti‐empathy. In A. Coplan, & P. Goldie (Eds.), *Empathy: Philosophical and psychological perspectives* (pp. 302–317). Oxford University Press, p. 302.

39 Ibid: 303.

40 Ibid.

41 Garden, R. E. (2007). The problem of empathy: Medicine and the humanities. *New Literary History, 38*(3), 551–567, 557.

42 Ibid: 557.

43 Ibid: 557.

44 Ibid: 560.

45 Smajdor, S., Stöckl, S., & Salter, C. (2011). The limits of empathy: Problems in medical education and practice. *Journal of Medical Ethics, 37*(6), 380–383, 382.

46 Garden, R. (2008). Expanding clinical empathy: An activist perspective. *Journal of General Internal Medicine, 24*(1), 122–125, 124.

47 Fricker, M. (2007). *Epistemic injustice. Power and the ethics of knowing*. Oxford University Press, p. 1.

48 Kidd, I. J., & Carel, H. (2017). Epistemic injustice and illness. *Journal of Applied Philosophy*, 34(2), 172–190, 176.

49 Carel, H., & Kidd, I. J. (2014). Epistemic injustice in healthcare: A philosophical analysis. *Medicine, Health Care and Philosophy, 17*, 529–540, 531.

50 Kidd & Carel, op. cit. note 48, p. 177.

51 Frank, A. (2010). *The wounded storyteller: Body, illness and ethics*. University of Chicago Press, p. 96.

52 Jamison, L. (2014). *The empathy exams*. Greywolf Press, p. 5. As cited in Bloom, op. cit. note 24.

53 Carel & Kidd, op. cit. note 49, p. 531.

